# Draft Genome Sequences of the Altered Schaedler Flora, a Defined Bacterial Community from Gnotobiotic Mice

**DOI:** 10.1128/genomeA.00287-14

**Published:** 2014-04-10

**Authors:** Michael J. Wannemuehler, Ann-Marie Overstreet, Doyle V. Ward, Gregory J. Phillips

**Affiliations:** a Department of Veterinary Microbiology and Preventive Medicine, College of Veterinary Medicine, Iowa State University, Ames, Iowa, USA; b Broad Institute of MIT and Harvard, Cambridge, Massachusetts, USA

## Abstract

The altered Schaedler flora (ASF) is a bacterial community that supports normal growth and development of gnotobiotic mice. We report here the draft genome sequences of the 8 bacteria that comprise the ASF.

## GENOME ANNOUNCEMENT

The importance of the gastrointestinal (GI) microbiota in the health of the host has been known for decades. Roles of the GI microbiota include nutrient acquisition, protection against pathogens, and immune system development. Recent studies have added to our understanding by providing new mechanistic insights into host-microbiota interactions (1, 2). A significant challenge to these studies moving forward is the high abundance and diversity of bacterial species that colonize the mammalian GI tract, which is dominated by members of the phyla *Bacteroidetes* and *Firmicutes* (3). Recently, there has been renewed interest in the gnotobiotic mouse model, represented by the altered Schaedler flora (ASF), for the study of how gut bacteria impact the host (4–10).

The ASF mouse model is derived from the work of Schaedler et al., who colonized germfree mice with a consortium of bacteria that originated from conventional mice (11). Motivated by efforts by the National Cancer Institute (NCI) to generate mice colonized with a standardized microbiota, the ASF was subsequently derived from the original Schaedler flora, which comprised eight bacterial species. The ASF also consists of eight separate bacterial species, which were isolated from Swiss outbred mice, and includes four species not present in the original community (12). The ASF has subsequently been characterized by 16S rRNA sequence analysis to better determine the phylogeny of the members (13) and by quantitative PCR to assess the abundance, stability, and spatial distribution throughout the GI tract (14, 15). To further develop the ASF model as a resource for gut microbiota studies, we have determined the genome sequences of each of the bacterial species, which represent the first genome sequences of a complete mammalian GI bacterial community.

Whole-genome shotgun sequencing was done using Illumina sequencing technology to generate draft sequences for the 8 ASF strains, as summarized in Table 1. Genomic sequence reads were generated on an Illumina HiSeq 2000 machine. Data consisted of two libraries: one 180-bp insert paired-end library (16) and a large-insert, robotically size-selected, 3- to 5-kbp jumping library (17).

**TABLE 1:** Genome features and accession numbers of the ASF bacteria

ASF no.	Taxonomy	Genome size (Mb)	GC (%)	Gene count	Contig count	*N*_50_ (kb)	Fold coverage	GenBank accession no.
ASF356	*Clostridium* sp.	2.91	30.91	2,799	31	209	143	AQFQ00000000.1
ASF360	*Lactobacillus* sp.	2.01	35.86	1,930	244	19	47	AQFR00000000.1
ASF361	*Lactobacillus murinus*	2.17	39.96	2,102	78	59.7	160	AQFS00000000.1
ASF457	*Mucispirillum schaedleri*	2.33	31.15	2,144	39	151	142	AYGZ00000000.1
ASF492	*Eubacterium plexicaudatum*	6.51	42.86	6,217	248	74.4	119	AQFT00000000.1
ASF500	*Firmicutes* bacterium	3.70	58.77	3,563	42	300	137	AYJP00000000.1
ASF502	*Clostridium* sp.	6.48	47.90	6,062	134	137	82	AQFU00000000.1
ASF519	*Parabacteroides* sp.	6.87	43.45	5,477	24	584	143	AQFV00000000.1

Genome consensus was built *de novo* using ALLPATHS-LG (18) with default parameters, except for *Lactobacillus* bacterium ASF360, for which Velvet was used due to lack of jumping libraries for ASF360. Original assembly consensus was improved and corrected for *Mucispirillum schaedleri* ASF457 and *Firmicutes* bacterium ASF500 using the Pilon assembly improvement tool (D. Ward, unpublished data). Assemblies were analyzed using the GAEMR (http://www.broadinstitute.org/software/gaemr/) assembly evaluation package and manually reviewed for quality.

Protein-coding genes were predicted with Prodigal (19) and filtered to remove genes with at least 70% overlap of tRNAs or rRNAs, which were identified using tRNAscan-SE (20) and RNAmmer (21), respectively. Gene product names were assigned based on top blast hits against the Swiss-Prot protein database (at least 70% identity and at least 70% query coverage) and a protein family profile search against the TIGRfam HMMER equivalogs. More detailed characterizations of the ASF genomes are forthcoming.

### Nucleotide sequence accession numbers.

This whole-genome shotgun project has been deposited at DDBJ/EMBL/GenBank under the accession numbers shown in Table 1.
